# Assessment of Brain Functional Activity Using a Miniaturized Head-Mounted Scanning Photoacoustic Imaging System in Awake and Freely Moving Rats

**DOI:** 10.3390/bios11110429

**Published:** 2021-10-30

**Authors:** Yuhling Wang, Tsung-Sheng Chu, Yan-Ren Lin, Chia-Hui Tsao, Chia-Hua Tsai, Tzong-Rong Ger, Li-Tzong Chen, Wun-Shaing Wayne Chang, Lun-De Liao

**Affiliations:** 1 Institute of Biomedical Engineering and Nanomedicine, National Health Research Institutes, Zhunan Township, Miaoli County 35053, Taiwan; yuhlingwang@nhri.edu.tw (Y.W.); tsungsheng_chu@cycu.org.tw (T.-S.C.); tsaochiahui@nhri.edu.tw (C.-H.T.); vanessatsai@nhri.edu.tw (C.-H.T.); 2 Department of Biomedical Engineering, College of Engineering, Chung Yuan Christian University, Chung Li District, Taoyuan City 32023, Taiwan; sunbow@cycu.edu.tw; 3 Department of Emergency and Critical Care Medicine, Changhua Christian Hospital, Changhua County 50006, Taiwan; 117214@cch.org.tw; 4 College of Medicine, National Chung Hsing University, Taichung 402, Taiwan; 5 National Institute of Cancer Research, National Health Research Institutes, Zhunan Township, Miaoli County 35053, Taiwan; leochen@nhri.edu.tw; 6 Kaohsiung Medical University Hospital, Kaohsiung Medical University, Sanmin District, Kaohsiung City 80708, Taiwan

**Keywords:** freely moving animals, in vivo imaging, photoacoustic (PA), fiber-bundle-based illumination, hemoglobin oxygen saturation

## Abstract

Understanding the relationship between brain function and natural behavior remains a significant challenge in neuroscience because there are very few convincing imaging/recording tools available for the evaluation of awake and freely moving animals. Here, we employed a miniaturized head-mounted scanning photoacoustic imaging (hmPAI) system to image real-time cortical dynamics. A compact photoacoustic (PA) probe based on four in-house optical fiber pads and a single custom-made 48-MHz focused ultrasound transducer was designed to enable focused dark-field PA imaging, and miniature linear motors were included to enable two-dimensional (2D) scanning. The total dimensions and weight of the proposed hmPAI system are only approximately 50 × 64 × 48 mm and 58.7 g (excluding cables). Our ex vivo phantom experimental tests revealed that a spatial resolution of approximately 0.225 mm could be achieved at a depth of 9 mm. Our in vivo results further revealed that the diameters of cortical vessels draining into the superior sagittal sinus (SSS) could be clearly imaged and continuously observed in both anesthetized rats and awake, freely moving rats. Statistical analysis showed that the full width at half maximum (FWHM) of the PA A-line signals (relative to the blood vessel diameter) was significantly increased in the selected SSS-drained cortical vessels of awake rats (0.58 ± 0.17 mm) compared with those of anesthetized rats (0.31 ± 0.09 mm) (*p* < 0.01, paired *t*-test). In addition, the number of pixels in PA B-scan images (relative to the cerebral blood volume (CBV)) was also significantly increased in the selected SSS-drained blood vessels of awake rats (107.66 ± 23.02 pixels) compared with those of anesthetized rats (81.99 ± 21.52 pixels) (*p* < 0.01, paired *t*-test). This outcome may result from a more active brain in awake rats than in anesthetized rats, which caused cerebral blood vessels to transport more blood to meet the increased nutrient demand of the tissue, resulting in an obvious increase in blood vessel volume. This hmPAI system was further validated for utility in the brains of awake and freely moving rats, showing that their natural behavior was unimpaired during vascular imaging, thereby providing novel opportunities for studies of behavior, cognition, and preclinical models of brain diseases.

## 1. Introduction

In the treatment of neurological and cognitive impairments caused by disease or brain injury, the first step is to understand the relationship between brain functional changes and behavior. Rodents are ideal model organisms used in cell and molecular research to assess natural movement behaviors related to brain functioning. Electrophysiological (EP) recordings and electroencephalogram (EEG) data can provide high temporal resolution brain activity monitoring [1,2]. For EP studies in freely moving rodents, chronically implanted electrodes are used for either wired [3,4] or, more recently, wireless techniques [5]. Although neuronal action potentials can be recorded in freely moving rodents, the spatial resolution of these potentials is limited and insufficient for a clear definition of the actual neural network due to sampling limitations and volume conduction [6]. A two-photon microscope (TPM) with fluorescence imaging support can provide high-temporal and spatial resolution images of neural activity [6,7]. Recent advances in TPM techniques enable stabilized recordings of neural activity in various moving substrates [8]. However, this method requires an invasive procedure and therefore has the disadvantage of damaging the blood-brain barrier (BBB), which exerts possibly unfavorable effects on neurons and vasculature, and the recordings are limited to the area of interest; thus, long-term research is affected by this approach [6].

Positron emission tomography (PET) and functional magnetic resonance imaging (fMRI) technologies can be applied for monitoring overall cerebral functional activity. However, the disadvantages are that PET has a relatively low spatial resolution of only millimeters, while fMRI has a relatively low temporal resolution of seconds [9]. Functional Doppler ultrasound (US) can monitor freely moving rats, and it can provide information about cerebral blood flow (CBF) changes with high temporal and spatial resolution [10]. However, observed CBF changes cannot signify the oxygen content of hemoglobin [11,12,13]. Fully understanding the dynamics of hemoglobin, which provides the earliest indication of local neuronal activities through hemodynamic changes, is important. Cutting-edge technology uses laser-induced photoacoustic imaging (PAI) optical contrast to provide a positive solution. Theoretically, when the absorption contrast undergoes an expansion and contraction process caused by thermal change during tissue absorption of pulsed light, a photoacoustic (PA) wave is generated [14]. US detection is much less affected than optical imaging by tissue scattering [15]; thus, PAI retains the required optical contrast and can be used to obtain deep-tissue images (up to 1 cm) with a spatial resolution as fine as the micrometer level. Recent studies have demonstrated PAI monitoring of in vivo cerebral hemodynamics [16,17,18]. Observing changes in hemodynamics in the cortical layer is the essential first step in revealing the sequence of responses at different depths in a specific brain area [6].

Here, we propose a miniaturized head-mounted scanning photoacoustic imaging (hmPAI) system that can image hemodynamic responses and be utilized to evaluate the cortical layer for brain research in awake and freely moving rats. The total size of the hmPAI system was only 50 mm × 64 mm × 48 mm and weighed 58.7 g, excluding cables. This system can not only detect real-time hemodynamic changes in the cortical layer but also provide brain B-scan images of the front and rear horizontal planes using a scanning PA probe with a 48-MHz US transducer and fiber-based illumination. The hmPAI system performed scans and used four linear motors to obtain 3D images, such as B-scan and C-scan images. The scanning step was 0.12 mm, and the maximum scanning area was 8 mm × 6 mm, with an entire scan period of 56 min. To validate the hmPAI system, signal-to-noise ratios (SNRs) were determined from in vitro imaging of a blue ink phantom. Finally, we tested the in vivo functional ability of the developed hmPAI system by probing (1) real-time cortical hemodynamic changes in the superior sagittal sinus (SSS) blood vessel by PA A-line imaging under anesthesia and awake conditions and (2) the real-time dynamics of cortical hemodynamic changes at different positions relative to the bregma by PA B-scan and C-scan imaging under anesthesia and awake conditions. Collectively, this alignment-free design concept of a compact hmPAI system is intended to meet the diverse needs of neuroscientists performing preclinical studies of the brain.

## 2. Materials and Methods

### 2.1. Dark-Field Miniature hmPAI System with Fiber-Bundle-Based Illumination

**Figure 1:**
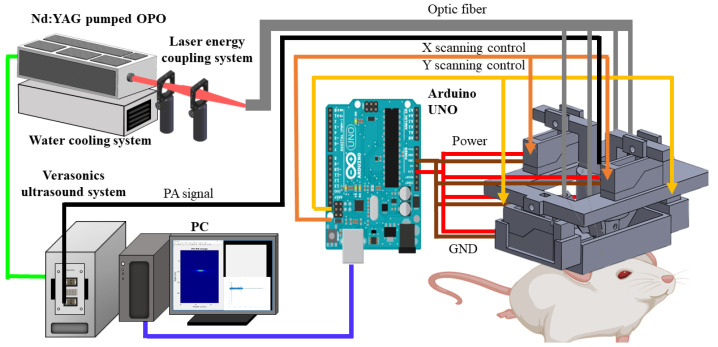
Diagram of the head-mounted hmPAI system with fiber-bundle-based illumination. First, the laser system provides laser pulses to the optical fiber through the lens, and the optical fiber is connected to the hmPAI device. The hmPAI device is mainly composed of four linear motors and controlled by the developed Arduino system. The holder was made by light-cured 3D printing. In the PC base, we used our own designed interface to control the entire imaging and scanning system.

Figure 1 shows a schematic of the hmPAI system, including the scanning control, laser illumination, PA/US signal acquisition, and experimental setup. Imaging and data acquisition were performed using a multichannel Verasonics high-frequency US platform (Vantage 128, Verasonics Inc., Kirkland, WA, USA). A custom graphical user interface (GUI) script was designed in MATLAB^®^ (R2007a, MathWorks Inc., Natick, MA, USA) to control the data acquisition sequence. For PA imaging, a trigger signal was used to synchronize the laser excitation and transducer data acquisition [19]. The signal was acquired using a large-numerical-aperture wideband 48-MHz ultrasound transducer [20] with a −6 dB fractional bandwidth of 57.5%, a 9 mm focal length, and a 6 mm active element [19]. For PA excitation, 750 nm or 800 nm light with a 7 ns duration and 20 Hz pulse repetition rate was generated using a tunable optical parametric oscillator (OPO) system (SpitLight 600 OPO, InnoLas Laser GmbH, Krailling, Germany) [19]. A-line signals were collected at 20 lines/s, and 10 A-line signals were averaged to decrease noise, resulting in a 2-line/s imaging rate.

**Figure 2:**
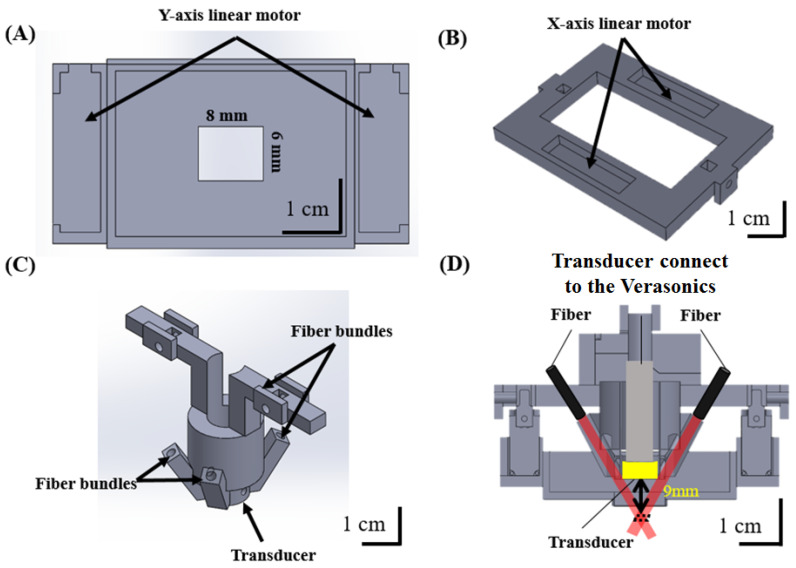
Diagram of the developed hmPAI system introducing each size, component, and assembly. (**A**) Holder for the *y*-axis linear motor. Black arrows indicate the positions of two *y*-axis motors. (**B**) Holder for the *x*-axis linear motor. Black arrows indicate the positions of the *x*-axis motors. (**C**) Holder for the 48 MHz transducer and fiber bundles. Black arrows indicate where the fiber bundles and transducer are placed. (**D**) Cross-sectional view of the hmPAI system after assembly.

The detailed design of the miniature head-mounted holder for the hmPAI system is shown in Figure 2. Four linear servo motors (VS-19, Solarbotics Ltd., Calgary, AB, Canada) controlled by an Arduino UNO controller (Arduino Corp., Boston, MA, USA) were used to move the transducer for scanning in the x- and y-directions (Figure 2A,B). The minimum step size was approximately 0.12 mm. The servo motors and PA probes were mounted on a 3D-printed holder with a hollow chamber in the center that was designed as a water tank to ensure efficient acoustic coupling during scanning (Figure 2A). In the PA imaging mode, laser light was delivered through the four output ends of a fiber bundle (Figure 2C) with angles adjusted to mimic dark-field illumination at the focal zone of the transducer (Figure 2D). The holders were designed in SolidWorks 2015 (Dassault Systèmes S.A., Vélizy-Villacoublay, France) and printed in ABS-like resin using a 3D printer (Shuffle 4k, Phrozen, Inc., Hsinchu City, Taiwan) with an accuracy of 0.03 mm. The delivered energy/pulse was 16–18 mJ.

### 2.2. Assessing the Spatial Resolution of the Developed hmPAI System

**Figure 3:**
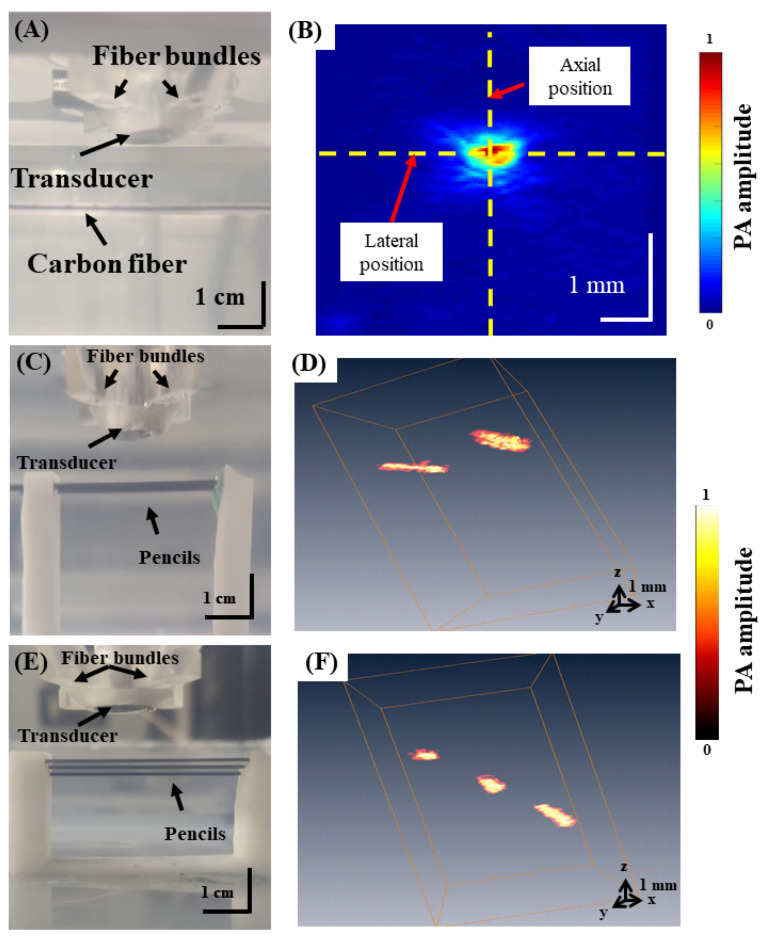
Performance tests for the axial and lateral resolutions of the developed hmPAI system using 79 µm carbon fiber and pencil lead phantoms. (**A**) Photograph of the in vitro carbon fiber phantom imaging experiment setup using the hmPAI system in a water tank. The size of the carbon fiber was approximately 79 µm, as measured by an LED handheld microscope. (**B**) PA B-scan image and axial resolution tests of the developed Arduino-based scanning system with steps of 0.12 mm at a depth of 9 mm, as quantified using a 79 µm carbon fiber phantom measured by FWHM. The results indicated that the spatial resolution of measurements by the Arduino-based scanning system was approximately 0.225 mm. (**C**) Performance tests of the developed hmPAI system using two pencil leads to simulate blood vessels at the same depth (i.e., 9 mm). (**D**) 3D PA image of the two pencil leads at the same depth measured by the developed Arduino-based scanning system (Movie S1 in Supplementary Materials). (**E**) Performance tests using three pencil leads at imaging depths of 8, 9, and 10 mm with the developed hmPAI system. (**F**) PA 3D image of three pencil leads at different depths produced by the developed Arduino-based scanning system (Movie S2).

Carbon fiber and pencil graphite were imaged to assess the spatial resolution of the hmPAI system. A carbon fiber of approximately 79 µm diameter, as measured by a light-emitting diode (LED) handheld microscope (UPG670 USB3.0, UPMOST, Taipei City, Taiwan), was imaged at a 9 mm depth and 750 nm wavelength in a water tank (Figure 3A). For B-scan imaging, a step size of 0.12 mm was used. The axial and lateral resolutions were determined as the full width at half maximum (FWHM) obtained from plotting the PA signal amplitude in the axial and lateral directions. Next, graphite pencil leads with a diameter of approximately 0.5 mm (Figure 3C,E) were imaged in a water tank. The pencil leads were placed at depths of 8, 9, and 10 mm from the transducer (Figure 3E,F) and imaged using an excitation laser with a wavelength of 750 nm [19]. For C-scan imaging, step sizes of 0.12 mm were used in both the x- and y-directions.

### 2.3. Craniotomy for Imaging Cortical Blood Vessels in Awake Animals

A total of 7 male Sprague Dawley rats (BioLASCO Taiwan Co., Ltd., Taipei City, Taiwan) weighing 250–350 g were used for cortical blood vessel imaging experiments, including 4 rats for the PA A-line and B-scan blood vessel diameter assessment experiments and 3 rats for the PA B-scan imaging at different locations with respect to the bregma. The experimental procedures were approved by the Institutional Animal Care and Use Committee of the National Health Research Institute (approved protocol numbers: NHRI-IACUC-107100-A and NHRI-IACUC-108044-M2-A). Craniotomy was performed under 1–3% isoflurane (Bowlin Biotech Corp., Taipei City, Taiwan) anesthesia. Using the bregma as the center, an 8 (horizontal) × 6 (vertical) mm cranial window was created with a high-speed drill [21].

## 3. Results

Despite its small size, the developed hmPAI system was able to provide good spatial resolution (0.225 mm) and probe rapid cerebral hemodynamic changes (2 lines/s for PA A-line signals) in awake and freely moving rats. We conducted a series of experiments (e.g., ex vivo phantom and in vivo rat imaging) to determine the capabilities of the system. Ex vivo phantom validation showed that the hmPAI system was able to achieve a spatial resolution of 0.225 mm. In vivo functional imaging ability tests were conducted by detecting cortical blood vessel diameter changes and cortical cerebral blood volume (CBV) changes in anesthetized and awake, moving rats using a wavelength of 800 nm. The dynamics of changes in the selected blood vessel diameter and its CBV were significantly larger in amplitude in awake and freely moving rats than in anesthetized rats. Additionally, based on PA C-scan data, there was a significant difference in regional hemodynamics between awake and freely moving rats and anesthetized rats at the same bregma position.

### 3.1. Imaging Performance of the Developed hmPAI System

A schematic diagram of the developed hmPAI system is shown in Figure 1. To facilitate device wearability for rat brain imaging, the developed system needs to be smaller in weight and size than the first developed scanning system. We designed a new holder for the four linear motors using light-cured 3D printing (Figure 2). To test the in vitro imaging performance of the hmPAI system, a carbon fiber phantom and a pencil lead phantom were imaged, as shown in Figure 3.

Figure 3B presents PA B-scan images of the spatial resolution test target (i.e., carbon fiber). The yellow dotted line represents data from the axial and lateral positions of the PA image (Figure 3B). The measured axial resolution was 0.225 mm. Figure 3D shows the C-scan image obtained from imaging pencil leads at 9 mm depth (Movie S1). Both pencil leads were successfully imaged with a good SNR. Figure 3F shows the PA 3D images obtained from imaging pencil leads at different depths (Movie S2). All three pencil leads were successfully imaged and could be differentiated. The SNR for the in vitro imaging tests was 33.41.

### 3.2. Setup Details of In Vivo Experiments Using the Developed hmPAI System

**Figure 4:**
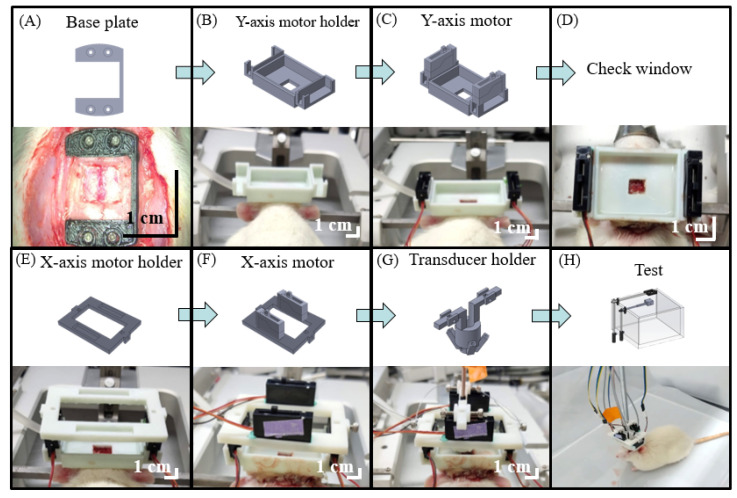
Surgical preparation procedure for the developed hmPAI system for the rat brain. (**A**) The base plate is used to set screws in the skull. (**B**) The *y*-axis motor bracket slides into the base plate through the track. (**C**) The *y*-axis motor is set in the *y*-axis motor holder with four screws. (**D**) The user checks the window to ensure that the surgical area is visible. (**E**) The *x*-axis motor holder is set in the *y*-axis motor with two screws. (**F**) The *x*-axis motor is set in the *x*-axis motor holder with four screws. (**G**) The transducer holder is set in the *x*-axis motor with two screws. (**H**) Test of the developed hmPAI system with awake rats in an acrylic box.

Figure 4 depicts the surgical procedure for the developed hmPAI system. After craniotomy, four holes were drilled, and four screws were used to lock the base plate. The y-axis motor holder was inserted into the base plate via an angled slot mechanism. In addition, a special glue stick was used to affix the two components to each other such that the hmPAI system would not easily fall apart during animal movement in awake experiments and to prevent motion artifacts. Two *y*-axis linear motors were placed in the *y*-axis motor holder, and four screws were used to lock the motor into the holder, as shown in Figure 4A–C. Figure 4D indicates that after skull-removal surgery, the location of the hmPAI system could be visualized. The two screws were used to secure the *x*-axis holder to a y-axis motor, and then two *x*-axis motors were placed in the *x*-axis motor holder using four screws. Two screws were used to make the transducer group with an *x*-axis motor. Figure 4E–H indicate the other steps executed to finish the preparation surgery, including the addition of the *x*-axis motors and transducer.

**Figure 5:**
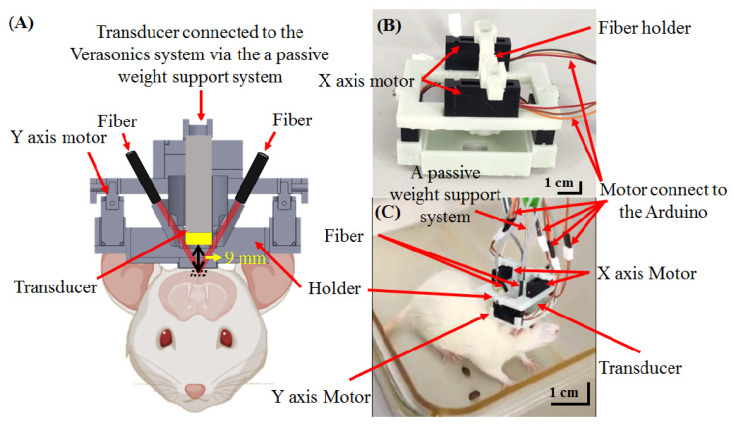
Schematic diagram of the cross-sectional view of the developed hmPAI system, describing all parts in detail, including the placement of the bracket, optical fiber, probe, *x*/*y*-axis motors, and Arduino device and the actual location on the rat. (**A**) Schematic showing the laser light path and transducer orientation. (**B**) Photograph of the assembled holder. (**C**) Photograph of the assembled system with transducer and fiber mounted on the head of a rat.

After the hmPAI system was set correctly for the rat brain, a schematic diagram showing a cross-sectional view of the hmPAI system was developed, as shown in Figure 5A. The interaural line and bregma were used to position the hmPAI system. Figure 5B shows a photograph of the complete hmPAI system with four linear motors. The movement speed of the linear motor used was set to 0.12 mm/s. In addition, a passive weight support system (Figure 5C) ensured that the rats were able to move freely while wearing the developed hmPAI device. All cables were suspended on the passive weight support system by a spring (elasticity k = 10 N/m) attached to a sliding linear bearing to enable the animal to move freely [22]. The hmPAI system was adjusted to approximately 40 mm height with transformed weights of ± 55 g at heights of 40 ± 12 mm. Generally, the height of the rat’s head remained within ±40 mm during experiments, indicating that the weight exerted on its head was less than 40 g. Thus, the support system reduced the weight imposed by the hmPAI system on the experimental animals and allowed good mobility during the experiment. With the reduced weight, animals would not feel overburdened and try to remove the hmPAI system. The photographs in Figure 5C show an experimental rat wearing the hmPAI system, and the mobility of the rat is demonstrated in Movie S3.

### 3.3. In Vivo Functional Imaging of Changes in Cortical Hemodynamics in SSS Blood Vessels

**Figure 6:**
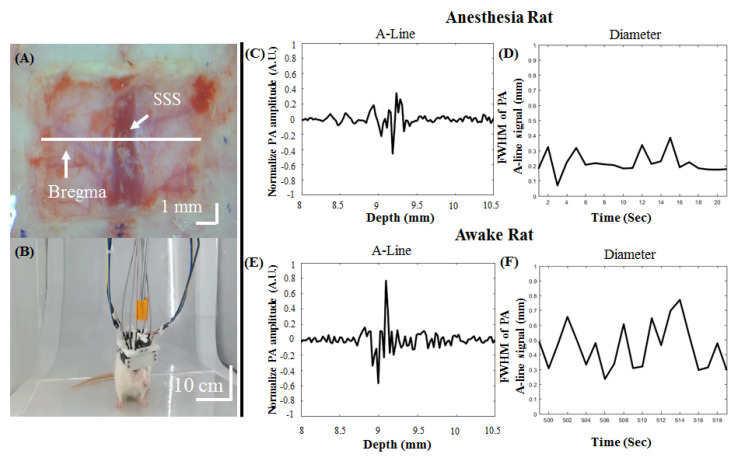
In vivo PA_800_ signals from diameter changes in cortical SSS blood vessels at the bregma position from awake and anesthetized rats. (**A**) Photograph of the rat brain after craniotomy for the addition of the developed hmPAI system. (**B**) Representative movie depicting awake and freely moving experimental rats wearing the hmPAI system (Movie S3). (**C**,**D**) Normalized and FWHM PA_800_ A-line cortical SSS blood vessel signals at bregma in anesthetized rats (Movie S4). The diameter of cerebral blood vessels in rats under anesthesia was approximately between 0.07 and 0.41 mm. (**E**,**F**) Normalized and FWHM PA_800_ A-line cortical SSS blood vessel signals at bregma in awake rats (Movie S4). In this awake rat study, the diameter of the selected SSS blood vessels of the rat ranged from 0.27 to 0.78 mm.

Figure 6 shows the in vivo PA_800_ signals from diameter changes in cortical SSS blood vessels at the bregma position in awake and anesthetized rats. A photograph of the rat brain was taken after craniotomy when it was ready for the addition of the developed hmPAI system, as shown in Figure 6A. The representative image in Figure 6B (Movie S3) depicts awake and freely moving experimental rats while wearing the hmPAI system.

**Figure 7:**
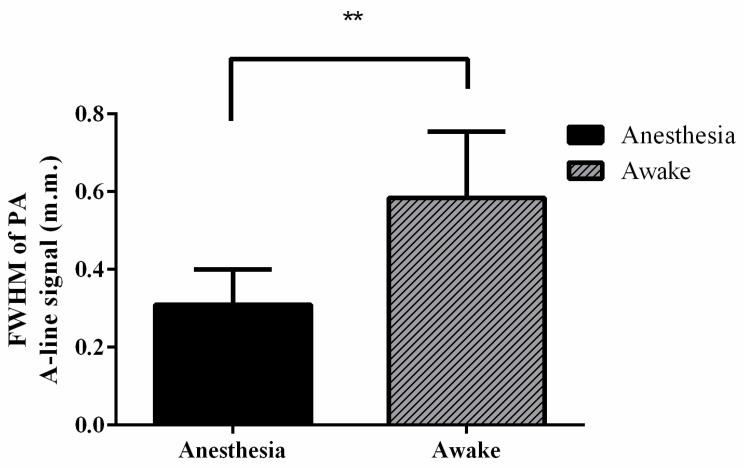
FWHM values of PA A-line signals in anesthetized (black) and awake (gray) rats. The diameter of the SSS blood vessel was significantly larger in the awake state than in the anesthetized state. This may be due to the continuous activity of awake rats, which caused their brains to be more active. Rat brains need more nutrients when the rats are active; that is, more blood needs to be delivered to the blood vessels in the brain, resulting in significant diameter changes. Error bars represent SD. ** *p* < 0.01 (Paired *t*-test, n = 4).

In vivo real-time changes in PA A-line signals (Figure 6C,E) and the corresponding FWHM signals (Figure 6D,F) from cortical SSS blood vessel diameter changes at the bregma position in anesthetized and awake rats were successfully recorded at various time points (Movie S4). The diameter of cerebral blood vessels in rats under anesthesia was approximately between 0.07 and 0.41 mm, and in the awake state, it ranged from 0.27 to 0.78 mm. The statistical analysis results are shown in Figure 7, which demonstrate that the FWHM values of the PA A-line signal under cortical SSS blood vessel changes in anesthetized and awake rats were 0.31 ± 0.09 mm and 0.58 ± 0.17 mm, respectively (*p* < 0.01, paired *t*-test, n = 4). Based on statistical analysis, the diameter changes in SSS blood vessels in awake rats were significantly larger than those in rats under anesthesia.

**Figure 8:**
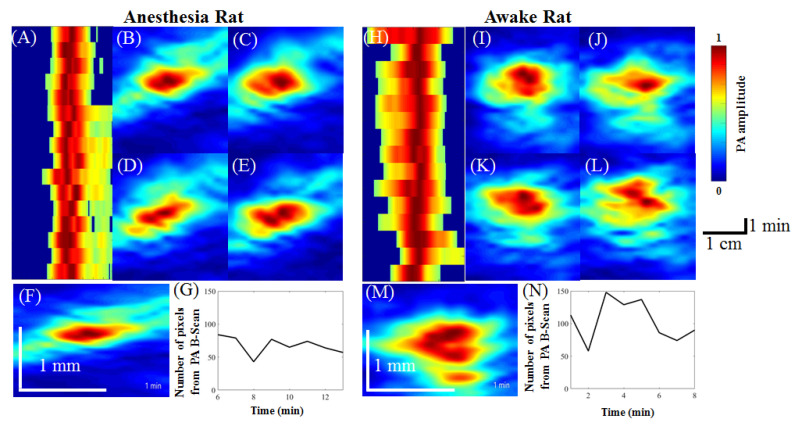
PA B-scan monitoring of the dynamics of the selected SSS blood vessel in anesthetized and awake rats. (**A**) PA MAP image of the time course of vessel diameter changes in anesthetized rats. The *x*-axis indicates the PA B-scan image, and the y-axis indicates the scanning time. The scanning time in this representative illustration is 16 min. (**B**–**E**) PA B-scan cross-sectional images of the target blood vessel at 1, 4, 9, and 14 min of anesthesia. (**F**,**G**) Video of the PA B-scan images and the number of pixels in PA B-scan images of anesthetized rats at different time points (16 min in total) (Movie S5). The number of pixels in the PA B-scan images of the measured SSS blood vessel ranged between 45 and 135. (**H**) PA MAP image of the time course (16 min) of blood vessel diameter changes in awake rats. The *x*-axis indicates the PA B-scan image, and the *y*-axis indicates the scanning time. The scanning time in this representative illustration is 16 min. (**I**–**L**) PA B-scan cross-sectional images of the target SSS blood vessel in an awake rat at 4, 6, 8, and 14 min. (**M**,**N**) PA B-scan images and the number of pixels in PA B-scan images of anesthetized and awake rats at different time points (16 min in total) (Movie S5). The number of pixels in the PA B-scan of the measured SSS blood vessel ranged between 58 and 148.

**Figure 9:**
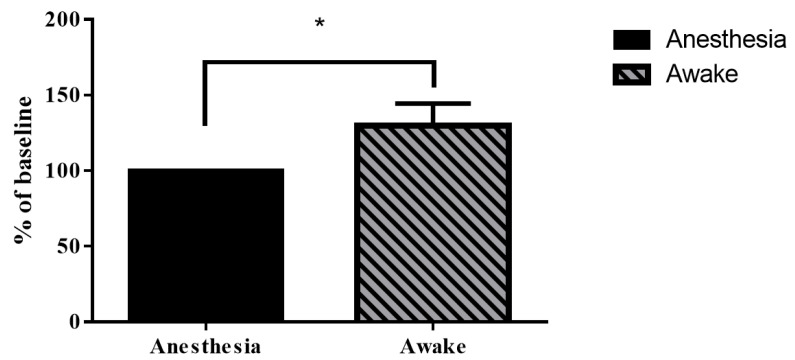
The percent of baseline blood vessel diameter from the PA B-scan image in anesthetized (black) and awake (gray) rats, with the anesthetized state as the baseline. There was a significant difference in the diameter of the blood vessels in the awake state compared to that in the anesthesia state. This result is in agreement with the result observed in the PA A-line. The reason may be that the brain is more active because awake rats exhibit continuous activity; thus, more nutrients must be transported by blood in the cerebral blood vessels, resulting in obviously increased blood vessel volume. Error bars represent SD. * *p* < 0.05 (Paired *t*-test, n = 3).

According to the optical absorption spectra of hemoglobin [23,24], changes in CBV can be detected by PA imaging using an excitation wavelength of 800 nm. Figure 8 shows the PA B-scan monitoring of CBV dynamics in anesthetized rats and awake rats. Figure 8A indicates the time course of the CBV changes in the anesthetized rats. Figure 8B–E present the B-scan images of selected SSS blood vessels at 1, 4, 9, and 14 min under anesthesia. The number of pixels in the PA B-scan images of the measured SSS blood vessel ranged between 45 and 135. In this experiment, the developed hmPAI system was used to observe whether the CBV changes in SSS blood vessels in awake and anesthetized rats were different. We first monitored changes in blood vessels in the brains of anesthetized rats using PA images, and we combined images from different time points into a video, with images shown in Figure 8F (Movies S5). Then, as shown in Figure 8G (Movies S5), we made a video of the number of pixel changes (i.e., CBV changes) from the PA B-scan images of the cerebral blood vessels at different time points to observe the CBV changes in the area of the blood vessels. Figure 8H shows the time course of CBV changes in awake rats. In Figure 8I–L, we show PA B-scan images of awake rats at 4, 6, 8, and 14 min to observe cerebral CBV changes in blood vessels. Next, we show the PA images of the SSS blood CBV changes at all time points in awake rats in Figure 8M (Movie S5). As shown in Figure 8N (Movie S5), we made a video showing the number of pixel changes in the PA B-scan images of the cerebral blood vessels at different time points to observe the CBV changes in the blood vessels of awake rats. Statistical analysis showed that the average % of baseline blood vessel diameter of awake rats was 129.8% (*p* < 0.05, paired *t*-test, n = 3) when compared to baseline anesthesia state, as shown in Figure 9. The pixel numbers from the PA B-scan image in this article represent the relative changes in CBV [20]. Based on statistical analysis, the relative changes in CBV of SSS blood vessels in awake rats were significantly larger than those in rats under anesthesia.

**Figure 10:**
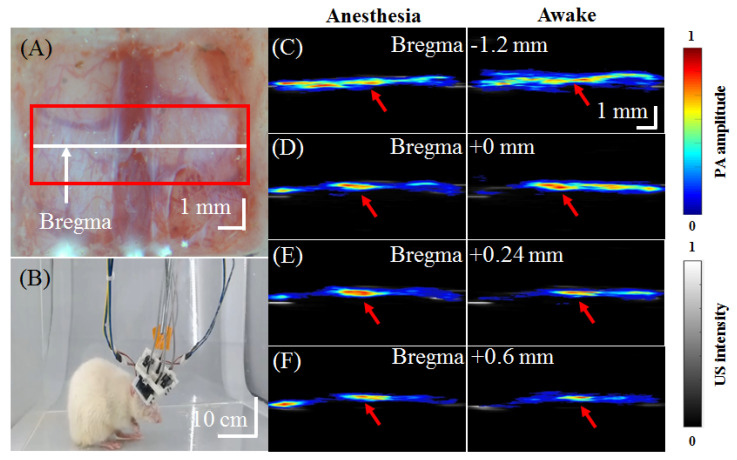
In vivo PA_800_ B-scan images of the cortical region of the rat brain at the positions of bregma −1.2, 0, +0.24, +0.6 mm in anesthetized and awake rats. (**A**) Photograph of the rat brain after craniotomy for addition of the hmPAI system. (**B**) Representative movie depicting awake and freely moving experimental rats equipped with a wearable hmPAI system (Movie S3) for PA B-scan imaging. (**C**–**F**) PA B-scan images at bregma −1.2, +0, +0.24, and +0.6 mm in anesthetized and awake rats. The experimental results show that the developed hmPAI system can successfully perform PA B-scan imaging at different brain positions.

In Figure 10A, a red box is used to mark the detection range of the hmPAI system in this experiment. After surgery, the hmPAI system was fixed on the rat brain. Then, ketoprofen (5 mg/kg) was administered to the rats subcutaneously as an analgesic, and time was allowed to elapse to ensure that the rat was awake. After the rat was awake, we started the imaging session. An experiment image is shown in Figure 10B. Figure 10C–F present four B-scan locations. For example, the hmPAI system collected both US and PA images at bregma −1.2 mm, bregma +0 mm, bregma +0.24 mm, and bregma +0.6 mm positions in both anesthetized (left) and awake (right) rats. The same US transducer obtained B-mode US images with the hmPAI system working in pulse-echo mode. Because of the strong acoustic reflection, little information could be collected below the dura surface from the US images. However, the PA images provided much cortical vasculature information beneath the dura. It is known that most of the dominant signal source comes from the cortical vasculature [25]. For example, an SSS-drained cortical blood vessel, as marked by a red arrow in the figure, was readily evident, indicating that the hmPAI system could reliably image targeted blood vessels at a depth of approximately 9 mm. The SNR of the in vivo experiments was 29.82.

## 4. Discussion

### 4.1. Measurement of Cerebral Hemodynamics in Anesthetized and Awake Rats Using the Developed hmPAI System

We conducted experiments on anesthetized and awake rats using the developed hmPAI system. Our results demonstrated that the developed hmPAI system successfully detected cerebral CBV and blood vessel diameter changes in the rat brain in anesthetized and awake rat models using 800 nm wavelength PA B-scan and C-scan imaging modes. The findings suggested that the hmPAI system could detect CBV and blood vessel diameter changes within the brain vasculature; therefore, the system will be applicable for measuring pathological changes in the near future [26]. In addition, our data indicated that rats in the awake and freely moving state showed (1) significantly larger blood vessel diameter dynamics and (2) significantly higher CBV intensity increments than rats in the anesthetized state. Our findings agree with previous reports [27,28] that CBV dynamics are relatively decreased in amplitude by anesthesia compared with the awake state.

Many in vivo animal models have used anesthesia when studying vascular dynamics. Although these models have greatly contributed to the current knowledge of vascular dynamics in various physiological processes, recent studies have found that anesthesia affects many physiological processes, including vascular dynamics. Therefore, caution should be taken in translating functional vascular imaging data acquired in anesthetized animal models to chronic diseases in humans. This study found that the CBV was obviously higher in awake animals than in those who received anesthesia. There were two possible reasons for this outcome. First, previous human-based studies indicated that most anesthetics decrease CBF or the metabolic rate of oxygen. Second, these agents influence cerebrovascular reactivity (related to CO_2_ responsiveness) and the regulation of neuronal hemostasis [29]. In addition, an animal under anesthesia or in a state of deep sleep (stage III/IV) requires less oxygenation and brain perfusion than animals in an awake state [30]. That is, the developed hmPAI system has demonstrated its utility for optical imaging in awake and freely moving animals, enabling us to address critical questions about vascular dynamics in a model that mimics awake humans.

### 4.2. Prospects of the Developed hmPAI System

Significant effort has been made to push the limitations of preclinical imaging techniques to resolve important issues in neuroscience, such as neurovascular communications, especially in the awake condition. Importantly, our results suggested that the hmPAI system could be used for imaging in awake and moving rodents; therefore, this system is an optimal tool that can be used to understand hemodynamic functions. Keeping an animal in an awake and freely moving state might be a relatively good way to document more realistic hemodynamic dynamics of cerebral vasculature in response to functional stimulation/tasks [28]. Additionally, more hemodynamic details, such as the vasodilatory response, intensity, and latency/recovery time, could be further revealed.

Functional photoacoustic microscopy (fPAM) can be used to obtain structural and functional information about cerebrovascular networks with good contrast and high spatial resolution [31]. However, current fPAM techniques cannot be applied in functional imaging studies of freely moving animals or provide concurrent B-scan and C-scan imaging of the same site [24,28]. The proposed hmPAI system is small and lightweight, has volumetric imaging capabilities, and is stable in the long term, thus enabling cerebral imaging of awake and freely moving animals. However, more improvements should be considered to investigate complicated brain activities/disorders and broaden the impact of this technique. First, the multiwavelength technique [32,33] and the PA/US Doppler effect [34] should be incorporated thus that additional functional parameters such as hemoglobin oxygen saturation and cerebral blood flow [33] can be measured. Second, for brain tumors, seizures, or stroke-like chronic brain diseases, an easily accessible cranial window for chronic imaging is preferred [35]. Third, to simultaneously study neural activities (i.e., electrocorticography) and hemodynamics, a reliable imaging/sensing technique to comprehensively assess neurovascular function is necessary [16,36].

The hmPAI system is an acoustic-resolution photoacoustic microscopy (AR-PAM) system [20] designed to mimic dark-field AR-PAM systems with decreased size and weight. Traditional dark-field AR-PAM systems with more complex lens systems can achieve resolutions of 45 µm [37]. For in vivo studies in awake and freely moving animals, decreasing the size and weight of the AR-PAM system is necessary, which can be accomplished by using fiber bundles and eliminating complex lens systems. Wen et al. [38] and Tang et al. [39] proposed compact systems that can be used for imaging awake and freely moving animals with resolutions of 500 µm and 243 µm, respectively. Our hmPAI system has a higher resolution of 225 µm, which can be improved by modifying the fiber bundle illumination design to resemble a donut shape.

Regarding the limitations of the system, the developed hmPAI system uses a 48 MHz frequency transducer, which is greater than ½ the sampling rate of the Verasonics system. To improve image quality and meet the Nyquist criteria, we plan to incorporate an interleaved imaging schematic [40] as the next step. In addition, the hmPAI is constrained to a single imaging plane, and movement of the PA probe for B- or C-scan imaging still relies on the scanning motor. Imaging applications that require larger fields of view (FOVs) often utilize other optical imaging methods, such as intrinsic optical imaging and laser speckle contrast imaging [41]. Examples include assessments of brain-wide hemodynamic dynamics in the cerebral cortex [41] or resting-state functional connectivity studies [42]. However, the developed hmPAI system can be modified to include miniature 2D matrix array transducers to resolve hemodynamic responses in different layers of the rat brain [43] or fast scanning strategies based on microelectromechanical system (MEMS) technology [44] that will enable comprehensive FOV imaging. This approach would extend the hmPAI system to larger regions, such as the whole brains of mice, rats, and rabbits. However, the main limitation of fast, volumetric imaging with a wide FOV generally arises from the abundance of real-time data collected and the number of reconstructed voxels that must be assessed when reconstructing time-resolved 3D datasets. Using a graphical processing unit (GPU) to accelerate reconstructions can be of help to individuals employing this approach and to display cerebral activities in real-time [45].

## 5. Conclusions

In this study, a miniature hmPAI system was developed using (1) a detachable fiber-bundle-based illumination structure integrated with (2) a 48-MHz high-frequency US transducer, and (3) a custom platform made by light-cured 3D printing. We successfully validated its US and PA performance in the brains of both anesthetized and awake freely moving rats. This approach takes advantage of dark-field illumination based on fiber bundles and a planar scanning mechanism using four linear servo motors controlled by an Arduino microcontroller. The total dimensions of the proposed hmPAI are only approximately 50 × 64 × 48 mm, and the total weight is approximately 58.7 g (excluding cables). It has a spatial resolution of approximately 0.225 mm. Our experimental results indicate that the dynamics of the diameters of selected SSS blood vessels in the cortical layer were significantly larger in awake rats than in anesthetized rats. With the developed hmPAI system, we can accurately detect an exciting phenomenon that illuminates both structural and functional dynamics of the cortex in awake, freely moving rats. In the future, the lateral resolution can be improved by (1) changing the mechanism design and (2) providing better dark-field lighting performance, which might enhance various parameters of imaging performance, ranging from the acoustic resolution (AR) to the optical resolution (OR). The developed hmPAI system can complement existing optical imaging techniques and offers a good in vivo tool for brain research using different experimental models, such as the ketamine addiction model, in freely moving awake rats.

## Data Availability

Data will be provided upon request by the corresponding author (Lun-De Liao) of this article.

## Supplementary Materials

The following are available online at https://www.mdpi.com/article/10.3390/bios11110429/s1: Movie S1: 3D PA image of 2 pencil leads at the same depth for Figure 3, Movie S2: 3D PA image of 3 pencil leads at different depths for Figure 3, Movie S3: awake and freely moving rat wearing the hmPAI system for Figure 6 and Figure 10, Movie S4: change in A-line signal and vessel diameter over time in awake vs. anesthetized rats for Figure 6, and Movie S5: PA B-scan image of SSS at different timepoints in awake vs. anesthetized rats for Figure 8.
